# Correlation of gasdermin B staining patterns with prognosis, progression, and immune response in colorectal cancer

**DOI:** 10.1186/s12885-024-12326-2

**Published:** 2024-05-06

**Authors:** Liang Sun, Jiahui Wang, Yuxuan Li, Yixin Kang, Yi Jiang, Jun Zhang, Senmi Qian, Fangying Xu

**Affiliations:** 1 Department of Pathology and Pathophysiology, Department of Hepatobiliary and Pancreatic Surgery of the Second Affiliated Hospital, Zhejiang University School of Medicine, 866 Yuhangtang Road, Hangzhou, Zhejiang 310058 P. R. China; 2 Key Laboratory of Disease Proteomics of Zhejiang Province, Zhejiang University School of Medicine, 866 Yuhangtang Road, Hangzhou, Zhejiang 310058 P. R. China; 3 Department of Statistics, School of Mathematical Sciences, Anhui University, Hefei, Anhui P.R. China

**Keywords:** Colorectal cancer, Prognosis, Immune microenvironment, Gasdermin B, Pyroptosis

## Abstract

**Background:**

Pyroptosis is a type of programmed cell death mediated by the gasdermin family. Gasdermin B (GSDMB), as a member of gasdermin family, can promote the occurrence of cell pyroptosis. However, the correlations of the GSDMB expression in colorectal cancer with clinicopathological predictors, immune microenvironment, and prognosis are unclear.

**Methods:**

Specimens from 267 colorectal cancer cases were analyzed by immunohistochemistry to determine GSDMB expression, CD3^+^, CD4^+^, and CD8^+^ T lymphocytes, CD20^+^ B lymphocytes, CD68^+^ macrophages, and S100A8^+^ immune cells. GSDMB expression in cancer cells was scored in the membrane, cytoplasm, and nucleus respectively. GSDMB^+^ immune cell density was calculated. Univariate and multivariate survival analyses were performed. The association of GSDMB expression with other clinicopathological variables and immune cells were also analyzed. Double immunofluorescence was used to identify the nature of GSDMB^+^ immune cells. Cytotoxicity assays and sensitivity assays were performed to detect the sensitivity of cells to 5-fluorouracil.

**Results:**

Multivariate survival analysis showed that cytoplasmic GSDMB expression was an independent favorable prognostic indicator. Patients with positive cytoplasmic or nuclear GSDMB expression would benefit from 5-fluorouracil based chemotherapy. The assays in vitro showed that high GSDMB expression enhanced the sensitivity of colorectal cancer cells to 5-fluorouracil. Patients with positive membranous or nuclear GSDMB expression had more abundant S100A8^+^ immune cells in the tumor invasive front. Positive nuclear GSDMB expression indicated more CD68^+^ macrophages in the tumor microenvironment. Moreover, GSDMB^+^ immune cell density in the stroma was associated with a higher neutrophil percentage but a lower lymphocyte counts and monocyte percentage in peripheral blood. Furthermore, the results of double immunofluorescence showed that GSDMB co-expressed with CD68 or S100A8 in stroma cells.

**Conclusion:**

The GSDMB staining patterns are linked to its role in cancer progression, the immune microenvironment, systemic inflammatory response, chemotherapeutic efficacy, and prognosis. Colorectal cancer cells with high GSDMB expression are more sensitive to 5-fluorouracil. However, GSDMB expression in immune cells has different effects on cancer progression from that in cancer cells.

**Supplementary Information:**

The online version contains supplementary material available at 10.1186/s12885-024-12326-2.

## Introduction

Cancer is a leading cause of death worldwide. Although the incidence and mortality of colorectal cancer (CRC) is declining in some countries owing to widespread colonoscopy screening and improved treatment, CRC still ranks third globally in morbidity (10.0%) and second in mortality (9.4%) [1]. Remarkable recent progress has been made in the field of cancer–microenvironment interaction, which has made a profound impact on the prognostic evaluation and treatment of cancer. Hence, gaining an in-depth understanding of the development of the great heterogeneity within cell types and their distribution in the tumor immune microenvironment (TIM) is urgently needed.

Pyroptosis was initially regarded as caspase-1-mediated cell death. However, recent studies have found that pyroptosis is executed by the gasdermin (GSDM) protein family, which can be cleaved in the linker between the amino-terminal GSDM-N and carboxy-terminal GSDM-C domains by caspase-1/3/7/8 and granzymes, etc [2–6]. . Pyroptosis has thus been redefined as GSDM-mediated programmed cell death in 2015 [7]. Generally, C-terminal domain of GSDMs is an inhibitory domain that prevents the N-terminal domain from transferring to the cell membrane and forming pores. Once liberated, the N-terminal domain oligomerizes and perforates the cell membrane to release the cellular contents and induce cell lysis [8]. The GSDM family consists of GSDMA, GSDMB, GSDMC, GSDMD, GSDME (or deafness, autosomal dominant 5, DFNA5), and DFNB59 (or pejvakin). All members have strong sequence similarities in their N- terminal regions.

GSDMB is distinct among all members of the GSDM family because it is the only one absent in the mouse and rat genomes [9]. Chao et al. [10] reported that both the full-length and N-terminal domain of GSDMB (GSDMB-N) could bind to the major lipid species (phosphoinositide [PI], and sulfatide) in the mammalian cell membrane. However, Hansen et al. [11] found that neither the full-length GSDMB nor GSDMB-N bound to PI and sulfatide, but the GSDMB-N interacted with cardiolipin, phosphatidylglycerol, and lipid A to induce pore formation. Furthermore, GSDMB could be cleaved by caspase-1/3/4/6/7/8/9 and granzyme A, among others [4, 12–14].

Intriguingly, as an executor of pyroptosis, many studies have reported that GSDMB expression is upregulated in cancer tissues [15–17]. In human epidermal growth factor receptor 2-positive breast cancer, GSDMB promotes metastasis, and indicates a poor prognosis and weaker therapeutic response [15, 18, 19]. In CRC, how is GSDMB expression correlated with various clinicopathological parameters? Does GSDMB expression affect the prognosis and efficacy of 5-fluorouracil (5-Fu) based chemotherapy?

Pyroptosis can be avoided by repairing damage in the plasma membrane [20]. Nonetheless, pores or prepores created by GSDM family mediate the release of pro-inflammatory factors, which induce strong immune reactions around the cell [21]. Contrary to many studies that suggesting that GSDMB forms pores in the cell membrane and causes pyroptosis [4, 11, 13, 22], two previous studies have shown that the overexpression of the GSDMB-N subunit failed to induce pore formation [14, 23].

In a study on the 4T1 mammary tumor graft, GSDMA3-mediated pyroptosis in less than 15% of tumor cells was sufficient to eliminate the entire tumor by inducing effective antitumor immunity [24]. Growing evidence has shown that innate immunocytes (macrophages, neutrophils, etc.) and adaptive immune cells (T and B cells) found in the tumor microenvironment contribute to tumor progression. Our previous study found that the density of S100A8^+^ cells in the tumor invasivefront (TIF) was an indicator of a good prognosis in CRC [25]. S100A8^+^ cells are mainly composed of the myeloid lineage including granulocytes and monocytes. GSDMB is involved in some immune diseases, such as asthma and inflammatory bowel disease [10], and is also expressed in immune cells [11, 14]. However, the relationship between GSDMB and the cancer immune microenvironment is unclear.

The release of pro-inflammatory intracellular contents during pyroptosis may initiate systemic inflammation. The association between GSDMB expression and systemic inflammation is still unknown. The amount and proportion of different immune cells in peripheral blood, the lymphocyte-to-monocyte ratio (LMR), neutrophil-to-lymphocyte ratio (NLR), platelet-to-lymphocyte ratio (PLR) and prognostic nutritional index (PNI, sum of albumin and lymphocyte counts) were considered representative of the systemic immune response in our study.

GSDMB is expressed broadly in gastrointestinal epithelia, and is located in membrane, cytoplasm, and nucleus [4, 17]. However, the clinical significance and function of GSDMB expression in different subcellular locations are still unclear. In this study, we evaluated the expression of GSDMB in the membrane, cytoplasm, and nucleus of cancer cells, as well as in the TIM, and assessed the distribution and density of some types of immune cells in 267 CRC samples with follow-up information. We further explored the association of GSDMB expression in different subcellular localizations with tumor progression, TIM, systemic inflammation, chemotherapeutic efficacy and overall survival.

## Methods

### Case materials

In all 267 cases with sporadic CRC that did not undergo preoperative chemotherapy or radiotherapy, 215 patients survived, whereas 52 were deceased at the end of follow-up. The follow-up period ranged from 1 to 153 months, with a median of 27 months and a mean of 40.6 months. One patient who died within 1 month after surgery was excluded from subsequent survival analyses. Of the 267 cases, 144 patients underwent regular chemotherapy based on 5-Fu after radical surgery, 121 never received chemotherapy, and 2 had unavailable data. This study was approved by the Ethics Committee of Zhejiang University School of Medicine.

### Clinicopathological parameters

Tumor location, age at diagnosis, and sex were retrieved from medical records. All archival slides stained with hematoxylin and eosin were reviewed. Two hundred specimens were adenocarcinoma not otherwise specified (NOS), whereas 67 were of other histological types, including mucinous adenocarcinoma, signet ring cell carcinoma, and undifferentiated carcinoma. Histological differentiation was graded into low grade (gland formation ≥ 50%, *n* = 188) and high grade (gland formation < 50%, *n* = 79). Vascular infiltration, perineural infiltration, infiltration depth, lymph node metastasis, distant metastasis, and TNM stage were also assessed.

### Systemic inflammatory indicators

Data on preoperative peripheral blood indicators were obtained from patient records. Data were also obtained on plasma albumin concentration, platelet count, total white blood cell count, neutrophil count, lymphocyte count, monocyte count, neutrophil percentage, lymphocyte percentage, monocyte percentage, and eosinophil percentage were involved in our study. PNI, LMR, PLR, and NLR were calculated. PNI was calculated as serum albumin (g/L) + 5 × total lymphocyte count × 10^9^/L. LMR was deemed as the ratio of the lymphocyte count to the monocyte count, PLR as that of the peripheral platelet count to the lymphocyte count, and NLR as that of the peripheral neutrophil count to the lymphocyte count.

### Tissue microarray

Tissue microarrays of the 267 colorectal cancer samples were constructed using archival formalin-fixed, paraffin-embedded blocks. Each case had three tissue perforations, namely, from normal mucosa, tumor center (TC), and TIF. The TC area was identified as at least a length of 20× field from the border of normal mucosa. The TIF area was identified as a length of 20× field within the farthest tumor cell. The 1 cm diameter punches were transferred to recipient paraffin blocks (6 × 7 punches). Finally, the recipient paraffin blocks were cut into 4-µm-thick slices for subsequent staining.

### Immunohistochemistry and double immunofluorescence staining

GSDMB antibody (ab215729, Abcam, Hangzhou, China) was a gift from Dr. F. Shao (National Institute of Biological Sciences, Beijing, China). CD3, CD4, CD8, CD20, CD68, and S100A8 antibodies were used to mark CD3^+^, CD4^+^, and CD8^+^ T lymphocytes, CD20^+^ B lymphocytes, macrophages, and S100A8^+^ immunocytes, respectively. CD3, CD4, CD8, CD20, and GSDMB were detected using tissue microarray, whereas CD68 and S100A8 were identified by staining whole-tissue slides. GSDMB expression was also evaluated in immune cells. Data on the primary antibodies and staining patterns are presented in Supplementary Table 1.

Briefly, sections were dewaxed and dehydrated, after which they underwent antigen repair, and finally, immunohistochemistry (IHC) or double immunofluorescene. Antigen retrieval was performed by microwave heating, and a two-step method (PV-9000 Polymer Detection System, Jinqiao, Zhongshan, Beijing, China) was used for IHC. 3, 3-diaminobenzidine (DAB) solution was used for color rendering followed by hematoxylin staining. As a negative staining control, slides were treated with 100mM pH 7.4 phosphate-buffered saline (PBS) solution instead of a primary antibody. If the tissues disintegrated during IHC, no data could be obtained. The NanoZoomer 2.0 HT (Hamamatsu, Japan) was used to digitally scan of all slides for IHC. GSDMB expression in cancer cells was assessed in the cytoplasm, membrane, and nucleus, respectively. Less than 1% of cancer cells with definitely brown staining were defined as negative, otherwise they were positive. A computer-automated method (Image-Pro Plus 6.0, Media Cybernetics Inc.) was used to count the number of immune cells in four hotspots respectively. Except for GSDMB^+^ immune cells which were counted under 40× high-power field (HPF), the other immune cells were counted under 20× HPF. The density of immune cells was calculated as average counts per HPF.

Double immunofluorescence staining was performed to detect the co-expression of GSDMB and other immune cell markers. After secondary antibody incubation, Opal 520 and Opal 570 (Akoya, MA, USA) were used for double immunofluorescence. GSDMB was stained in green fluorescence, whereas CD3, CD4, CD8, CD20, CD68, and S100A8 were visualized in red fluorescence. The images were captured with a fluorescence microscope (BX63 Olympus microscope, Olympus, Tokyo, Japan).

### Immunofluorescence assay

After discarding the culture medium, cells were fixed in 4% paraformaldehyde for 20 min and permeabilized with 0.5% Triton X-100 for 10 min at room temperature. Then, cells were blocked with 10% BSA in PBS for 1 h and incubated with primary antibody against GSDMB (1:500, ab215729, Abcam, Hangzhou, China) at 4 °C overnight, incubated with Alexa Fluor 546 AffiniPure Goat anti-rabbit secondary antibodies (1:500, A-11010, ThermoFisher, Shanghai, China) for 1 h at room temperature. Subsequently, the nuclei were stained with 4,6-diamidino-2- phenylindole (DAPI, 1:5000, Beyotime, Shanghai, China) for 20 min. The images were captured with a confocal microscope (OLYMPUS IX83-FV3000-OSR) at a magnification of 60×.

### Cell culture and reagents

HCT8 and SW480 cells were purchased from the American Type Culture Collection (VA, USA). HCT 8 and SW480 were cultured in RPMI 1640 supplemented with 10% fetal bovine serum. All cell lines had no mycoplasma contamination, and were identified using short tandem repeat-based methods. Cells were treated with 500µM of 5-Fu (Sigma-Aldrich, St. Louis, MO, USA) for 48 h for cytotoxicity assays.

### Plasmids, siRNA and transfection

The GSDMB overexpression pLVX-IRES-Zsgreen1 lentivirus plasmids were kindly provided by Dr. F. Shao. siGSDMB was used to knockdown GSDMB. The siGSDMB sequences were 5′-GCCUUGUUGAUGCUGAUAGAUTT-3′ for siGSDMB-1, and 5′-GCUGUAUGUUGUUGUCUCUAUTT-3′ for siGSDMB-2. Lipofectamine 2000 transfection reagent (Invitrogen, Carlsbad, CA, USA) was used for the plasmid transfection of eukaryotic cells. GenMute (SignaGen, Frederick, MD, USA) transfection reagent was used for siRNA knockdown.

### Western blotting

In vitro cultured cells were lysed in radioimmunoprecipitation assay lysis buffer (Beyotime, Shanghai, China) containing protease inhibitor mixture PMSF (Beyotime, Shanghai, China) and phosphatase inhibitor cocktail (MCE, NJ, USA). The GSDMB expression levels were detected by western blotting with GSDMB antibody (1:1000, ab215729, Abcam, Hangzhou, China). Tubulin (1:5000, T5201, Sigma-Aldrich, St. Louis, MO, USA) was used as control. Blots were then incubated with IR-dye secondary antibodies and visualized by Odyssey Imager (LI-COR, NE, USA).

### Cytotoxicity assays

Cell cytotoxicity was tested by the LDH assay using Cyto Tox 96 Non-Radioactive Cytotoxicity Assay kit (Promega).

### Sensitivity assays

5 × 10^3^ Cells were grown in 96-well plates and treated for 48 h with 5-Fu at concentrations of 0, 6.4, 32, 160, 800 and 4000 µM. After 48 h, the viability of cells was measured by CCK8 Assay. Curves were calculated on the basis of the absorbance readings collected from the CCK8 assay relative to drug concentrations. Absorbance was normalized to the vehicle controls, and drug concentrations were converted to logarithms by using GraphPad Prism (GraphPad Software, San Diego, CA). The half-inhibitory concentration (IC50) was determined as a 50% loss of viability occurring at this concentration compared to untreated cells.

### Statistical analysis

IBM SPSS Statistics 26.0 (IBM SPSS, Armonk, NY, USA) and GraphPad Prism (GraphPad Software, San Diego, CA) were used to perform statistical analyses. McNemar’s test was performed to test the differences in the proportions of GSDMB expression in the different locations. GSDMB expression was compared with other clinicopathological variables using chi-square or Fisher’s exact test. We used the t-test to analyze the differences in the means between two groups. Univariate survival analyses were performed with the “Survfit” function in R, and survival curves were drawn using the Kaplan-Meier method with a log-rank test. The life-table method was used to calculate the 5-year survival rate. The Cox proportional hazards model was used in multivariate survival analysis. A *P* value < 0.05 was defined as a significant difference. A statistical trend was identified if 0.05 < *P* value < 0.1.

## Results

### Expression profile of GSDMB in cancer cells

**Fig. 1:**
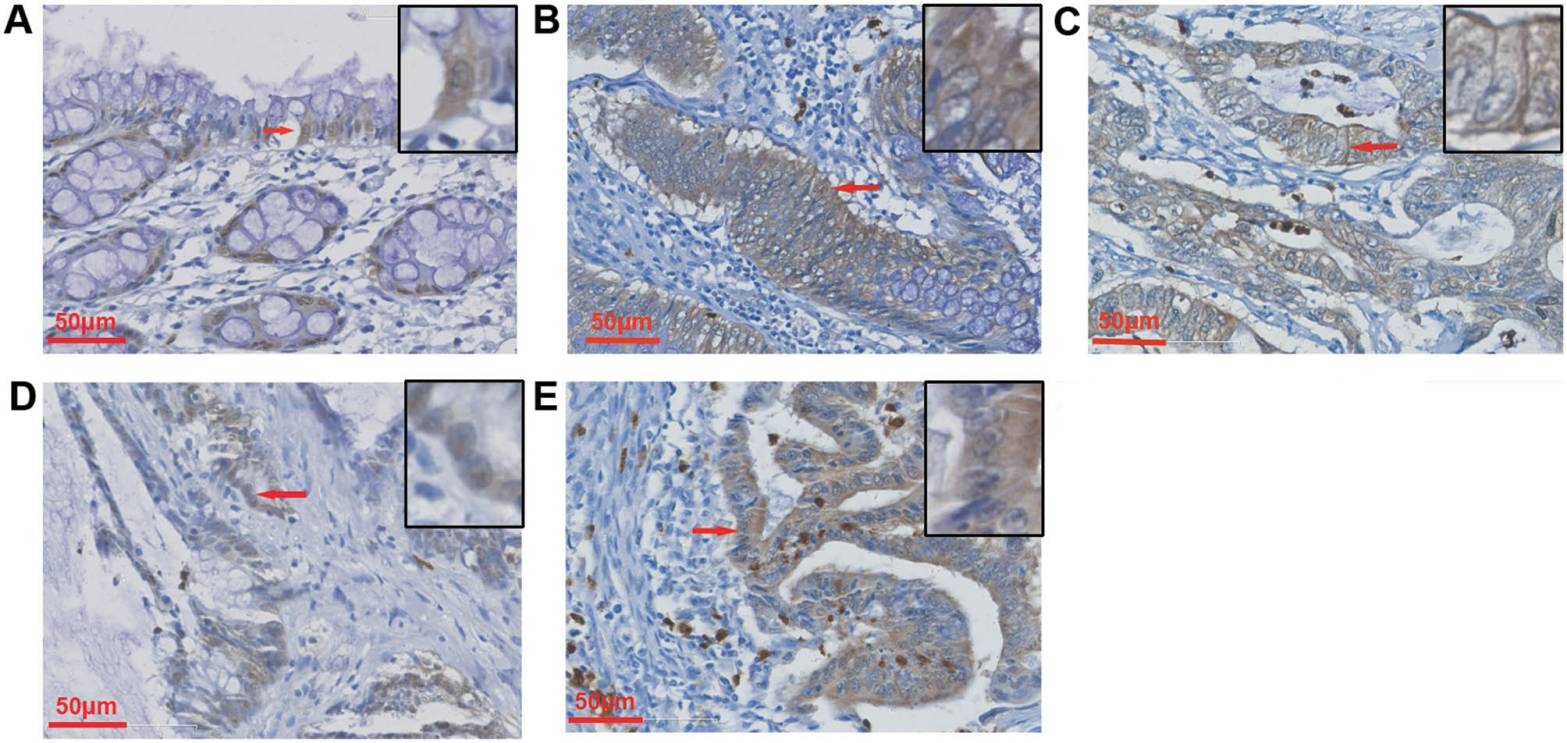
IHC images of GSDMB in normal epithelial cells (**A**); and in cytoplasm (**B**), membrane (**C**) and nucleus (**D**) of cancer cells; as well as in tumor invasive front (**E**). The representative cells are indicated with arrows. 400× magnification

GSDMB was expressed in the cytoplasm, membrane, and nucleus in both normal epithelial cells and cancer cells (Fig. 1A-1E). Positive GSDMB expression was defined as score > 0, whereas negative GSDMB expression was score = 0. The positive GSDMB expression rates in the cell membrane, cytoplasm and nucleus were 27.3% (73/267), 79.8% (213/267), and 67.8% (181/267), respectively, in cancer cells. In normal epithelial cells, the positive expression rates were 26.6% (50/188), 80.9% (152/188), and 86.7% (163/188), respectively (Supplementary Table 2). The rate of positive nuclear GSDMB expression in normal epithelial cells was higher than that in cancer cells.

### GSDMB expression in cancer cells and clinicopathological parameters

The results showed that positive membranous GSDMB expression was more common in cases with lower TNM stage and without lymph node metastasis (Table 1). The rate of positive cytoplasmic GSDMB expression was relatively higher in adenocarcinoma NOS than in other histological types. Those cases with cytoplasmic GSDMB expression were more likely to have a lower histological grade and less likely to present with vessel infiltration. No correlations were found between cytoplasmic GSDMB expression and age, sex, location, perineural infiltration, infiltration depth, lymph node metastasis, distant metastasis, or TNM stage. A positive nuclear GSDMB expression indicated a lower histological grade and a lower likelihood of lymph node metastasis. No significant differences were found between nuclear GSDMB expression and other clinicopathological indicators. We also analyzed the relationship between clinical pathological indicators and total GSDMB expression, regardless of subcellular localization, and the results showed that total GSDMB expression was correlated with lower histological grade (Supplementary Table 3). In summary, the expression of GSDMB is associated with low tumor invasiveness.

**Table 1:** Association between GSDMB expression in cancer cells and clinicopathological indicators

Clinicopathological indicators	Number	Membranous GSDMB expression (%)		*P* value	Cytoplasmic GSDMB expression (%)		*P* value	Nuclear GSDMB expression (%)		*P* value
		Negative	Positive		Negative	Positive		Negative	Positive	
Age				0.625			0.526			0.843
≤ 60 years old	94	70(36.1)	24(32.9)		21(38.90)	73(34.3)		31(36.0)	63(34.8)	
> 60 years old	173	124(63.9)	49(67.1)		33(61.1)	140(65.7)		55(64.0)	118(65.2)	
Sex				0.654			0.516			0.717
Male	144	103(53.1)	41(56.2)		27(50.0)	117(54.9)		45(52.3)	99(54.7)	
Female	123	91(46.9)	32(43.8)		27(50.0)	96(45.1)		41(47.7)	82(45.3)	
Location				0.980			0.483			0.515
Colon	135	98(50.5)	37(50.7)		25(46.3)	110(51.6)		41(47.7)	94(51.9)	
Rectum	132	96(49.5)	36(49.3)		29(53.7)	103(48.4)		45(52.3)	87(48.1)	
Histologic type				0.594			0.009*			0.465
Adenocarcinoma NOS	200	147(75.8)	53(72.6)		33(61.1)	167(78.4)		62(72.1)	138(76.2)	
Others	67	47(24.2)	20(27.4)		21(38.9)	46(21.6)		24(27.9)	43(23.8)	
Histological grade				0.630			0.007*			0.006*
Low	188	135(69.6)	53(72.6)		30(55.6)	158(74.2)		51(59.3)	137(75.7)	
High	79	59(30.4)	20(27.4)		24(44.4)	55(25.8)		35(40.7)	44(24.3)	
Perineural infiltration				0.616			0.556			0.720
Absent	184	132(68.0)	52(71.2)		39(72.2)	145(68.1)		58(67.4)	126(69.6)	
Present	83	62(32.0)	21(28.8)		15(27.8)	68(31.9)		28(32.6)	55(30.4)	
Vessel infiltration				0.183			0.060			0.509
Absent	229	163(84.0)	66(90.4)		42(77.8)	187(87.8)		72(83.7)	157(86.7)	
Present	38	31(16.0)	7(9.6)		12(22.2)	26(12.2)		14(16.3)	24(13.3)	
Infiltration depth				0.487			0.797			0.784
Within serosa	90	63(32.5)	27(37.0)		19(35.2)	71(33.3)		28(32.6)	62(34.3)	
Outside serosa or muscle	177	131(67.5)	46(63.0)		35(64.8)	142(66.7)		58(67.4)	119(65.7)	
Lymph node metastasis				0.076			0.643			0.092
Absent	141	96(49.5)	45(61.6)		27(50.0)	114(53.5)		39(45.3)	102(56.4)	
Present	126	98(50.5)	28(38.4)		27(50.0)	99(46.5)		47(54.7)	79(43.6)	
Distant metastasis				0.381			0.894			0.782
Absent	241	177(91.2)	64(87.7)		49(90.7)	192(90.1)		77(89.5)	164(90.6)	
Present	26	17(8.8)	9(12.3)		5(9.3)	21(9.9)		9(10.5)	17(9.4)	
TNM stage				0.079			0.991			0.387
I	57	40(20.6)	17(23.3)		12(22.2)	45(21.1)		15(17.4)	42(23.2)	
II	78	51(26.3)	27(37.0)		15(27.8)	63(29.6)		22(25.6)	56(30.9)	
III	106	86(44.3)	20(27.4)		22(40.7)	84(39.4)		40(46.5)	66(36.5)	
IV	26	17(8.8)	9(12.3)		5(9.3)	21(9.9)		9(10.5)	17(9.4)	

Significant *P* values are indicated with asterisks

### GSDMB expression in cancer cells and systemic inflammatory indicators

Compared with the negative membranous GSDMB group, the positive membranous GSDMB group had a higher percentage of eosinophils in peripheral blood (Table 2). Moreover, a positive nuclear GSDMB expression indicated a higher lymphocyte percentage and tended to be associated with a higher albumin concentration, lower platelet count, and higher PNI. No relationship was found between cytoplasmic GSDMB expression and any systemic inflammatory indicators. The GSDMB expression in cancer cells is associated with systemic inflammatory response.

**Table 2:** Association between GSDMB expression and systemic inflammatory indicators

Peripheral blood indicators	Membranous GSDMB		*P* value	Cytoplasmic GSDMB		*P* value	Nuclear GSDMB		*P* value
	Negative	Positive		Negative	Positive		Negative	Positive	
Albumin (g/L)	37.46 ± 0.47	36.6 ± 0.87	0.381	37.12 ± 0.82	37.29 ± 0.47	0.865	36.1 ± 0.83	37.69 ± 0.47	0.085
Platelet (×10^9^/L)	232.75 ± 7.39	238.33 ± 11.61	0.705	242.16 ± 15.93	231.87 ± 6.69	0.500	255.21 ± 14.31	226.09 ± 6.64	0.069
White blood cell (×10^9^/L)	6.66 ± 0.21	6.73 ± 0.36	0.867	6.75 ± 0.34	6.66 ± 0.21	0.836	6.74 ± 0.38	6.66 ± 0.20	0.852
Neutrophil (×10^9^/L)	4.56 ± 0.20	4.54 ± 0.39	0.972	4.64 ± 0.35	4.53 ± 0.21	0.803	4.65 ± 0.37	4.51 ± 0.21	0.729
Lymphocyte (×10^9^/L)	1.45 ± 0.05	1.53 ± 0.08	0.413	1.41 ± 0.08	1.49 ± 0.05	0.450	1.36 ± 0.08	1.51 ± 0.05	0.100
Monocyte (×10^9^/L)	0.45 ± 0.02	0.50 ± 0.04	0.179	0.45 ± 0.03	0.46 ± 0.02	0.801	0.46 ± 0.03	0.46 ± 0.02	0.813
Eosinophil (×10^9^/L)	0.16 ± 0.01	0.20 ± 0.03	0.106	0.22 ± 0.03	0.15 ± 0.01	0.100	0.21 ± 0.03	0.15 ± 0.01	0.128
Neutrophil percentage	0.66 ± 0.01	0.65 ± 0.01	0.513	0.67 ± 0.01	0.66 ± 0.01	0.445	0.67 ± 0.01	0.65 ± 0.01	0.352
Lymphocyte percentage	0.24 ± 0.01	0.24 ± 0.01	0.887	0.23 ± 0.01	0.24 ± 0.01	0.446	0.22 ± 0.01	0.25 ± 0.01	0.048*
Monocyte percentage	0.068 ± 0.002	0.071 ± 0.004	0.554	0.065 ± 0.003	0.070 ± 0.002	0.280	0.067 ± 0.003	0.070 ± 0.002	0.510
Eosinophil percentage	0.026 ± 0.002	0.036 ± 0.005	0.022*	0.032 ± 0.004	0.027 ± 0.002	0.280	0.032 ± 0.005	0.026 ± 0.002	0.173
PLR	202.70 ± 16.40	183.78 ± 18.26	0.549	191.22 ± 16.39	200.26 ± 16.35	0.780	226.14 ± 21.10	187.42 ± 16.47	0.191
NLR	4.07 ± 0.41	3.52 ± 0.53	0.490	3.68 ± 0.38	4.01 ± 0.42	0.691	3.95 ± 0.46	3.94 ± 0.43	0.991
LMR	3.86 ± 0.19	3.67 ± 0.34	0.645	3.71 ± 0.34	3.84 ± 0.19	0.744	3.80 ± 0.39	3.82 ± 0.18	0.946
PNI	44.74 ± 0.62	44.53 ± 1.02	0.867	44.48 ± 1.04	44.75 ± 0.62	0.838	43.05 ± 0.99	45.32 ± 0.62	0.056

The number in table is Mean of peripheral blood indicators ± SE

Significant *P* values are indicated with asterisks

### Correlation between GSDMB expression in cancer cells and TIM

**Fig. 2:**
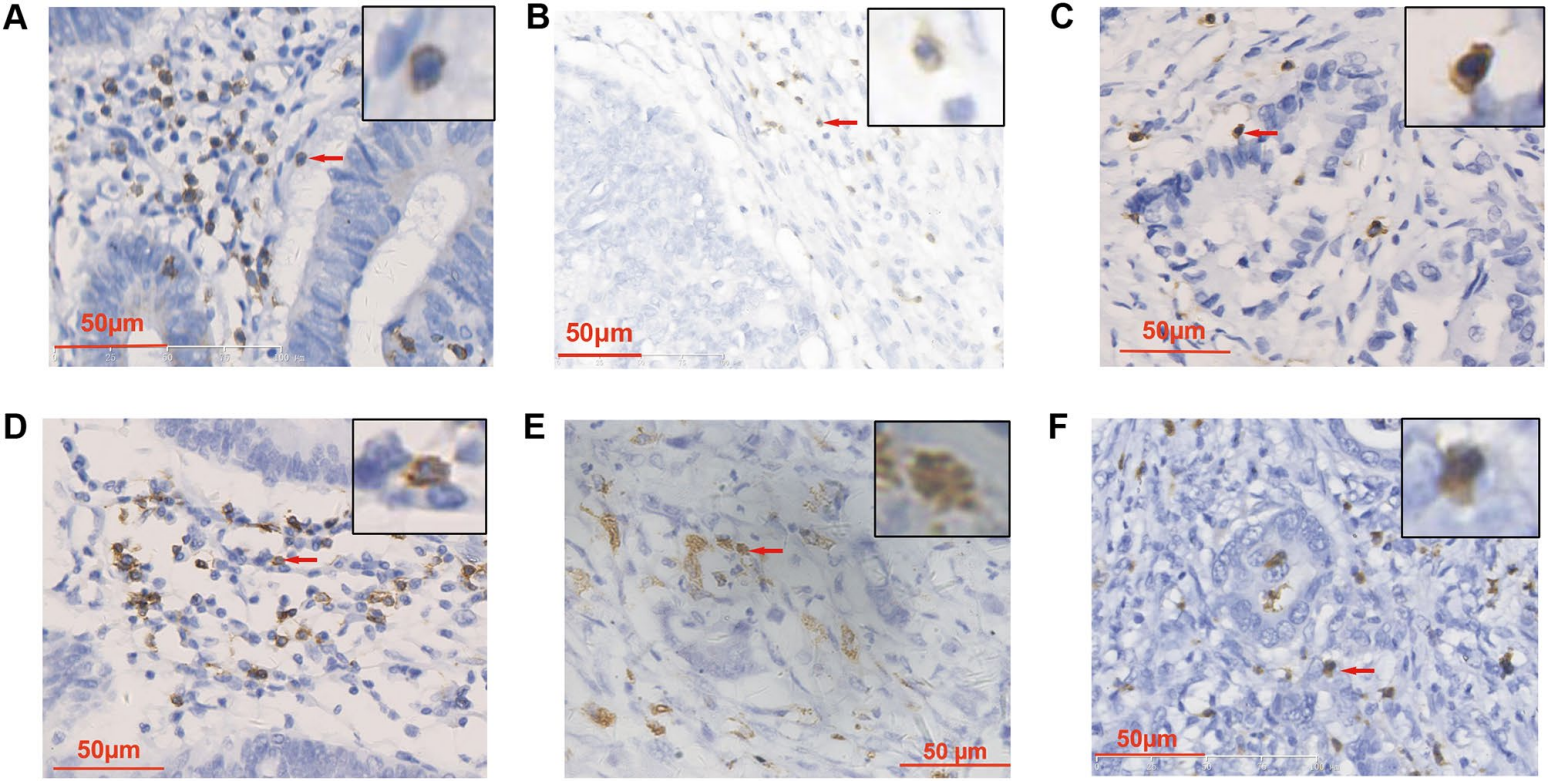
IHC images of CD3^+^ lymphocytes (**A**), CD4^+^ lymphocytes (**B**), CD8^+^ lymphocytes (**C**), CD20^+^ lymphocytes (**D**), CD68^+^ macrophages (**E**), and S100A8^+^ immune cells (**F**). The representative cells are indicated with arrows. 400× magnification

CD3^+^ lymphocytes, CD4^+^ lymphocytes, CD8^+^ lymphocytes, CD20^+^ lymphocytes, CD68^+^ macrophages, and S100A8^+^ immune cells were determined by IHC (Fig. 2). The density of each type of immune cells infiltrating the TIM was calculated in the TC and TIF separately. The distribution of S100A8^+^ immune cells in the TIF was more abundant in the positive membrane and nuclear GSDMB expression groups than in the respective negative groups (Table 3). Positive nuclear GSDMB expression was also correlated with a higher number of CD68^+^ macrophages in the TIM. However, cytoplasmic GSDMB expression was not correlated with either CD68^+^ macrophages or S100A8^+^ immune cells. Furthermore, no correlations were found between GSDMB expression in any subcellular localization with CD3^+^/CD4^+^/CD8^+^ T lymphocytes or CD20^+^ B lymphocytes. The expression of GSDMB in cancer cells is related to myeloid derived inflammatory cells rather than lymphocytes in TIM.

**Table 3:** Distribution and density of immune cells in TIM based on GSDMB expression in different subcellular localizations

Immune cells in TIM	Membranous GSDMB		*P* value	Cytoplasmic GSDMB		*P* value	Nuclear GSDMB		*P* value	
	Negative	Positive		Negative	Positive		Negative	Positive		
CD68^+^ macrophages (TIM )	64.08 ± 2.66	66.27 ± 4.63	0.670	64.57 ± 5.44	64.72 ± 2.55	0.979	57.05 ± 4.10	68.34 ± 2.76	0.022*	
CD68^+^ macrophages (TC)	25.81 ± 1.10	26.65 ± 2.03	0.700	26.33 ± 2.15	25.97 ± 1.09	0.879	24.64 ± 1.66	26.72 ± 1.19	0.316	
CD68^+^ macrophages (TIF)	43.31 ± 5.46	39.62 ± 3.12	0.681	55.88 ± 18.51	38.82 ± 1.82	0.363	43.52 ± 11.71	41.68 ± 1.97	0.831	
CD3^+^ T-lymphocytes (TIM )	48.34 ± 3.60	48.23 ± 5.50	0.987	50.53 ± 7.05	47.84 ± 3.34	0.734	53.36 ± 5.42	46.09 ± 3.61	0.267	
CD3^+^ T lymphocytes (TC)	17.50 ± 1.57	21.61 ± 2.91	0.185	18.77 ± 3.45	18.65 ± 1.53	0.974	19.34 ± 2.64	18.37 ± 1.65	0.749	
CD3^+^ T lymphocytes (TIF)	29.94 ± 2.72	26.31 ± 3.81	0.472	29.51 ± 5.34	28.82 ± 2.47	0.904	32.05 ± 4.13	27.56 ± 2.66	0.354	
CD4^+^ T lymphocytes (TIM)	4.54 ± 0.60	3.77 ± 0.73	0.488	4.67 ± 1.14	4.25 ± 0.54	0.725	4.50 ± 0.88	4.27 ± 0.59	0.826	
CD4^+^ T lymphocytes (TC)	1.93 ± 0.29	1.84 ± 0.33	0.871	1.89 ± 0.59	1.91 ± 0.25	0.968	1.98 ± 0.48	1.87 ± 0.27	0.820	
CD4^+^ T lymphocytes (TIF)	2.45 ± 0.38	1.91 ± 0.53	0.450	2.69 ± 0.68	4.84 ± 0.35	0.534	2.45 ± 0.53	2.25 ± 0.38	0.760	
CD20^+^ B lymphocytes (TIM )	11.67 ± 1.35	11.42 ± 1.82	0.921	10.99 ± 1.43	11.77 ± 1.35	0.776	11.98 ± 2.60	11.44 ± 1.09	0.821	
CD20^+^ B lymphocytes (TC)	3.07 ± 0.38	3.76 ± 0.89	0.408	4.28 ± 0.87	2.99 ± 0.40	0.156	3.38 ± 0.66	3.19 ± 0.44	0.803	
CD20^+^ B lymphocytes (TIF)	11.67 ± 1.35	7.68 ± 1.56	0.825	6.47 ± 1.11	8.46 ± 1.18	0.403	8.23 ± 2.34	7.97 ± 0.92	0.898	
CD8^+^ T lymphocytes (TIM )	24.33 ± 1.34	21.94 ± 2.00	0.340	26.37 ± 2.72	23.01 ± 1.22	0.233	25.34 ± 2.03	22.87 ± 1.34	0.303	
CD8^+^ T lymphocytes (TC)	8.82 ± 0.78	8.63 ± 1.12	0.895	9.98 ± 1.50	8.46 ± 0.71	0.343	9.16 ± 1.12	8.58 ± 0.78	0.671	
CD8^+^ T lymphocytes (TIF)	14.89 ± 0.87	12.96 ± 1.45	0.254	15.05 ± 1.80	14.21 ± 0.82	0.654	15.81 ± 1.42	13.72 ± 0.87	0.193	
S100A8^+^immune cells (TIF)	55.31 ± 4.22	75.77 ± 8.53	0.019*	49.97 ± 6.61	63.33 ± 4.52	0.172	49.70 ± 5.68	66.03 ± 5.00	0.032*	

The number in table is Mean of immune cell density ± SE

TIM: tumor immune microenvironment, TC: tumor center, TIF: tumor invasive front

Significant *P* values are indicated with asterisks

### Univariate survival analyses of GSDMB in cancer cells

**Fig. 3:**
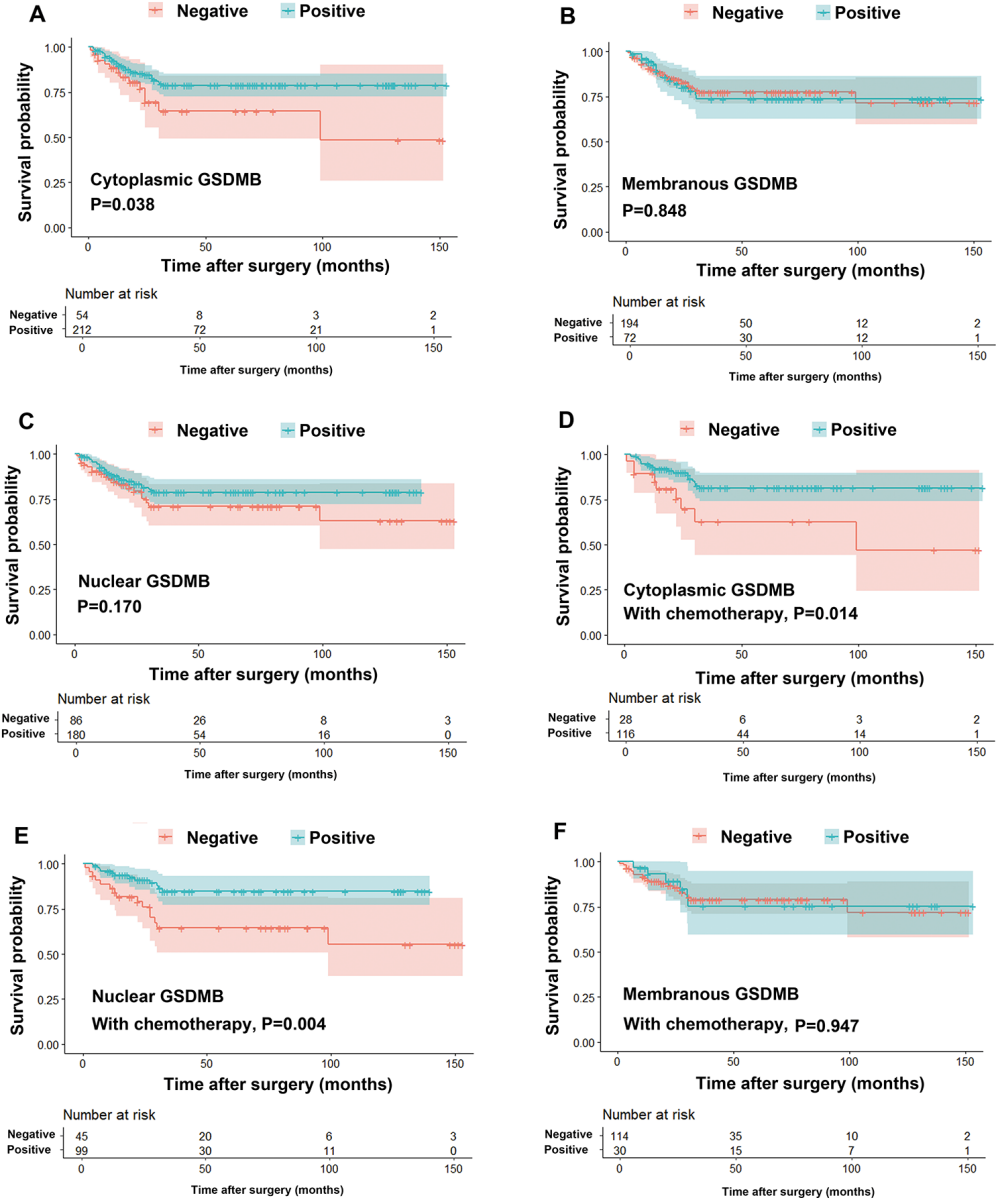
Survival curves of GSDMB expression in cytoplasm (**A**), membrane (**B**), and nucleus (**C**). Next, in group with chemotherapy after surgery, survival curves are plotted according to cytoplasmic GSDMB expression (**D**), nuclear GSDMB expression (**E**), and membranous GSDMB expression (**F**)

At the end of follow-up, 14 out of 54 patients died in the negative cytoplasmic GSDMB group and 37 out of 212 patients in the positive group died. Survival analyses for the different subcellular localizations of GSDMB expression showed that patients with positive cytoplasmic GSDMB expression had a better prognosis (Fig. 3A). The 5-year overall survival rate of the group with a positive cytoplasmic GSDMB expression was 78%, which was higher than the 63% of the group with negative cytoplasmic GSDMB expression. However, positive membranous or nuclear GSDMB expression had no correlation with overall survival (Fig. 3B and C).

The patients were further divided into two groups according to whether they underwent postoperative 5-Fu based chemotherapy. In the group that underwent chemotherapy, both cytoplasmic and nuclear GSDMB expression were favorable predictors of overall survival (Fig. 3D and E), whereas membranous GSDMB expression was not correlated with overall survival (Fig. 3F). GSDMB expression in any subcellular localization had no effect on overall survival in the cases without chemotherapy (Supplementary Fig. 1A–C).

**Fig. 4:**
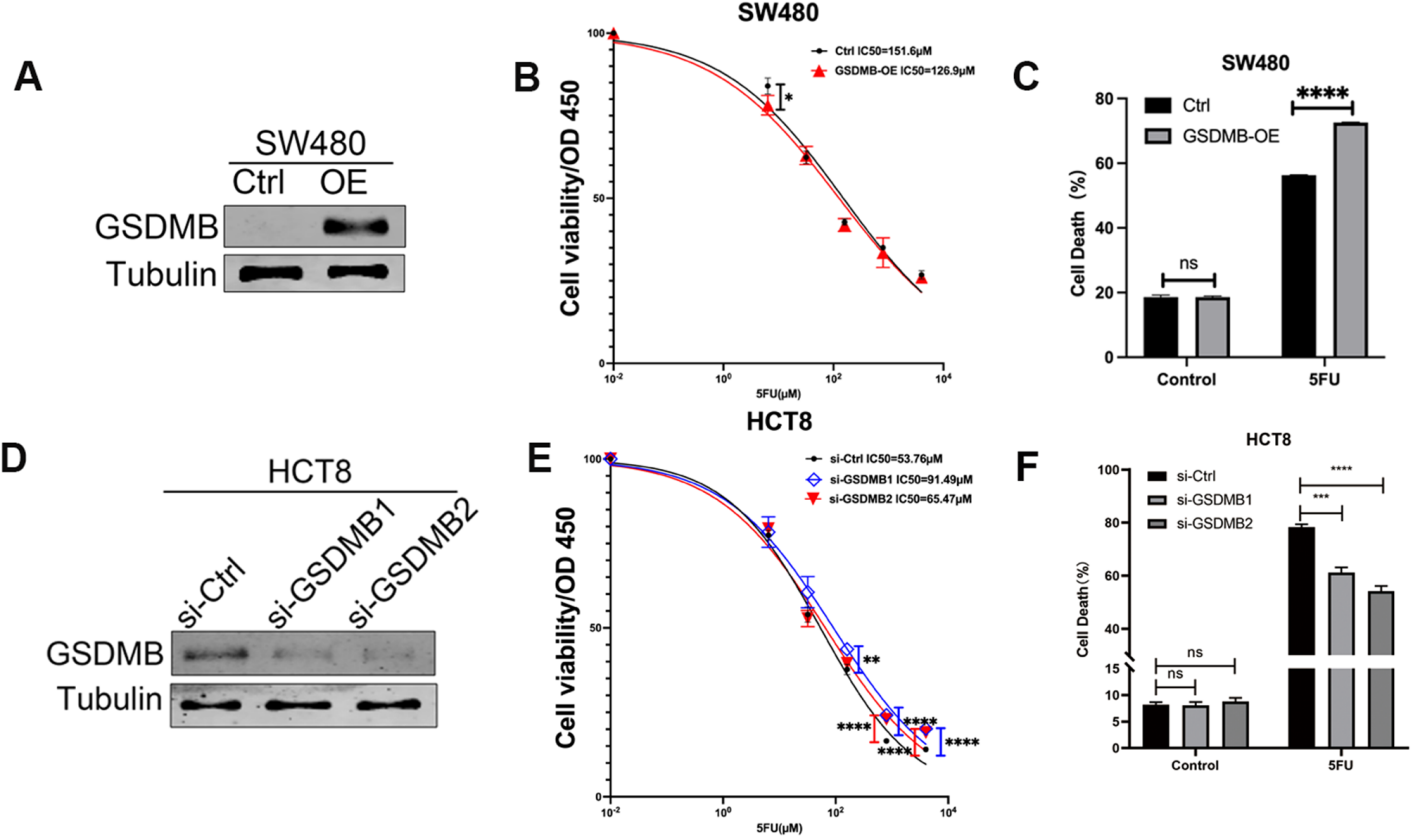
GSDMB regulates 5-Fu-induced lytic cell death. GSDMB was overexpressed in SW480 cells (**A**). Under 5-Fu treatment, GSDMB overexpression enhanced the SW480 sensitivity to 5-Fu (**B**) and induced cell death (**C**). GSDMB was knockdown in HCT8 cells (**D**). Under 5-Fu treatment, GSDMB knockdown represses HCT8 sensitivity to 5-Fu (**E**) and decreased cell death (**F**). For GSDMB and Tubulin bonds from different part of the same blot and different blots were combined. Molecular weight of analyzed proteins: GSDMB—50 kDa, Tubulin—55 kDa. Full-length blots are presented in Supplementary Fig. 4. Data are means ± s.d. *P < 0.05, **P < 0.01, ***P < 0.001, **** *P* < 0.0001, ns: no significant

To clarify the association between GSDMB expression and 5-Fu sensitivity, sensitivity assays and cytotoxicity assays were performed. GSDMB was overexpressed in the SW480 CRC cell line (Fig. 4A, Supplementary Fig. 2A). Sensitivity assays showed that the IC50 of 5-Fu in GSDMB overexpression cells and control were 126.9µM and 151.6µM respectively (Fig. 4B). Cytotoxicity assays showed that GSDMB overexpression upregulated LDH release and promoted lytic cell death (Fig. 4C). Moreover, GSDMB was knocked down in the HCT8 cell line, which has relatively high expression of GSDMB (Fig. 4D, Supplementary Fig. 2B). The IC50 results showed that knockdown of GSDMB significantly decreased the 5-Fu sensitivity of CRC cells (Fig. 4E). GSDMB knockdown repressed lytic cell death induced by 5-Fu (Fig. 4F). In general, the CRC patients with GSDMB expression are more likely to benefit from 5-Fu treatment.

### Cytoplasmic GSDMB is an independent favorable prognostic factor

Multivariate survival analysis was performed using the Cox proportional hazards model using age, sex, histological type, histological grade, vessel infiltration, perineural infiltration, TNM stage, chemotherapy, and cytoplasmic GSDMB expression. The results showed that TNM stage and cytoplasmic GSDMB expression were independent prognostic factors (Table 4). Cytoplasmic GSDMB expression indicated improved overall survival.

**Table 4:** The results of multivariate Cox proportional hazards model

Variable		RR (95%CI)	*P* value
Cytoplasmic GSDMB expression	Negative		
	Positive	0.476(0.250–0.909)	0.024*
TNM stage			< 0.001*
	I		
	II	1.546(0.517–4.618)	0.435
	III	2.613(0.989–6.902)	0.053
	IV	10.727(3.830-30.038)	< 0.001*

Significant *P* values are indicated with asterisks

### GSDMB^+^ immune cells in the TIF

**Fig. 5:**
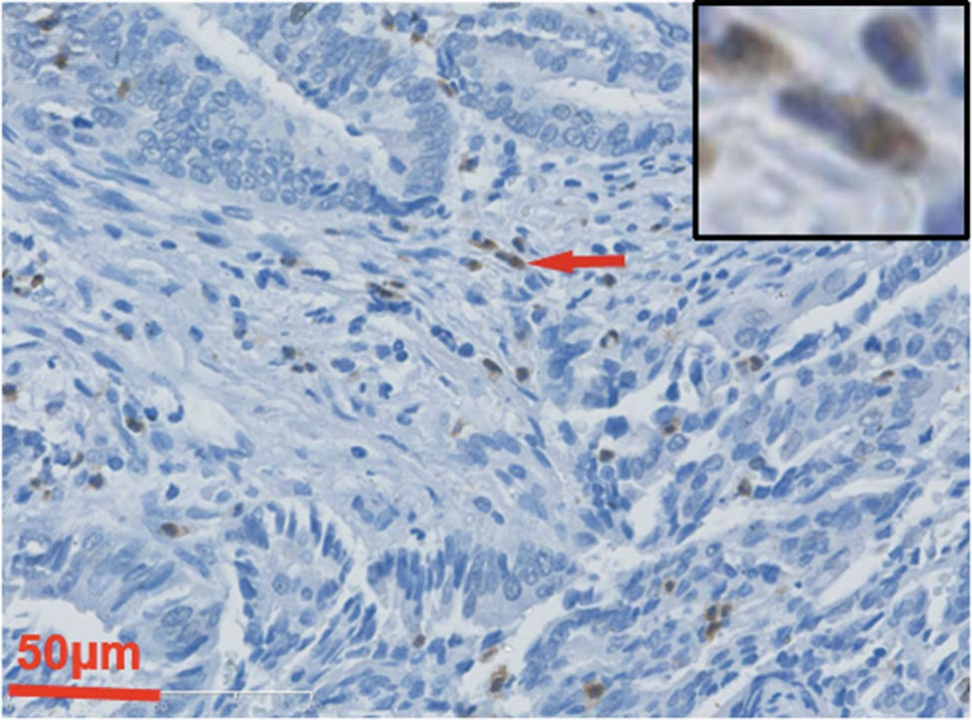
IHC images of GSDMB in immune cells. The representative cells are indicated with arrows. 400× magnification

In addition to tumor cells, we found that some immune cells also expressed GSDMB (Fig. 5). Therefore, the densities of GSDMB^+^ immune cells in TIF were calculated, which ranged from 0 to 120 (median: 10; mean: 18.01). GSDMB^+^ immune cell densities were divided into two groups (low and high) based on the mean. Men and patients aged < 60 years showed more GSDMB^+^ immune cells in the TIF (Supplementary Table 4). Among the parameters of systemic inflammatory indicators, high GSDMB^+^ immune cell density was linked to low lymphocyte count/percentage, low monocyte count/percentage, and high neutrophil percentage (Table 5). Univariate survival analysis showed that GSDMB^+^ immune cell density in the TIF was not correlated with overall survival. At the end of follow-up, 12/84 (14.3%) patients died in the group with high GSDMB^+^ immune cell density, whereas 24/140 (17.1%) patients died in the low-density group (Supplementary Fig. 1D).

**Table 5:** Association between the GSDMB^+^ immune cell density in TIF and Peripheral blood indicators

Peripheral blood indicators	GSDMB^+^ immune cells in TIF		*P* value
	Low	High	
Albumin (g/L)	37.28 ± 0.55	37.15 ± 0.70	0.888
Platelet (×10^9^/L)	234.12 ± 8.67	238.10 ± 9.69	0.767
White blood cell (×10^9^/L)	6.85 ± 0.27	6.43 ± 0.25	0.293
Neutrophil (×10^9^/L)	4.56 ± 0.25	4.52 ± 0.26	0.914
Lymphocyte (×10^9^/L)	1.54 ± 0,05	1.36 ± 0.06	0.027*
Monocyte (×10^9^/L)	0.49 ± 0.02	0.43 ± 0.02	0.090
Eosinophil (×10^9^/L)	0.16 ± 0.02	0.17 ± 0.02	0.657
Neutrophil percentage	0.64 ± 0.01	0.68 ± 0.01	0.046*
Lymphocyte percentage	0.25 ± 0.01	0.23 ± 0.01	0.096
Monocyte percentage	0.074 ± 0.003	0.065 ± 0.003	0.031*
Eosinophil percentage	0.027 ± 0.002	0.027 ± 0.003	0.974
PLR	179.83 ± 12,42	207.40 ± 17.20	0.191
NLR	3.67 ± 0.38	4.11 ± 0.62	0.528
LMR	3.84 ± 0.21	3.65 ± 0.24	0.571
PNI	45.11 ± 0.68	43.93 ± 0.90	0.299

The number in table is Mean of peripheral blood indicators ± SE

PLR: platelet-to-lymphocyte ratio, NLR: neutrophil-to-lymphocyte ratio, LMR: lymphocyte-to-monocyte ratio, PNI: prognostic nutritional index

To explore GSDMB expression in immune cell subtypes, double immunofluorescence staining was performed. The results suggested that GSDMB was co-expressed with CD68 or S100A8, but not with CD3, CD4, CD8, or CD20 (Supplementary Fig. 3). Therefore, we speculated that GSDMB^+^ immune cells may be derived from macrophages and neutrophils, and the GSDMB^+^ immune cell density correlate to the systemic inflammatory response.

## Discussion

Pyroptosis initiated by the GSDM protein family is a response to the primary immune reaction after pro-inflammatory stimulation. GSDMB is distinguished from other members of the GSDM family by its absence in rodents and its ability to bind to phospholipids as a full-length protein [10, 26]. These features indicate that GSDMB probably evolved exclusively in humans with some unknown function. In our study, we found that the different subcellular localization of GSDMB were associated with diverse cancer progression and immune response.

Zhou et al. [4] reported GSDMB expression in 75 hospital CRC samples and 155 commercial CRC samples. In the hospital samples, the positive rates of total GSDMB expression in cancer and adjacent normal tissue were 90.7% (68/75) and 96.0% (72/75) respectively, which is similar to ours (84.6% and 91.5%, respectively, data not shown). However, in commercial samples, the positive rates of GSDMB expression were 63.2% (98/155) in cancer and 52.9% (82/155) in adjacent normal tissue, which were much lower than those for their hospital samples and our samples. Moreover, Sun et al. [17] demonstrated by IHC a 72.7% (16/22) positive nuclear GSDMB expression rate in uterine cervix cancer samples and 33.3% (5/15) in normal samples, showing a similar positive rate of nuclear GSDMB expression in cancer tissues to ours (67.8%) but a lower positive rate in normal tissues. GSDMB mRNA expression was upregulated in gastric and breast cancers; however, no significant difference was found between cancer and stromal cells [15, 16].

Carl-McGrath et al. [27] found that GSDMB was expressed in the apical and luminal surfaces of normal colonic mucosa and crypt epithelial cells, as well as in apically oriented CRC. Saeki et al. [28] reported that GSDMB was expressed in the esophageal basal cell layer and in the isthmus/neck of the stomach. In our study, we found that GSDMB was expressed in whole cells, including the cytoplasm, membrane, and nucleus. Furthermore, the location of GSDMB was correlated with prognosis, but only cytoplasmic GSDMB expression is an independent favorable prognostic factor in CRC.

Intriguingly, many studies have suggested that GSDMB is an unfavorable prognostic indicator, but our research showed that cytoplasmic GSDMB expression indicates an improved prognosis in CRC. GSDMB expression is correlated with a shorter survival time and increased metastasis in breast cancer [15]. In HER-2–positive breast cancer, about 65% cases show GSDMB gene overexpression, which is linked to poor clinical outcomes, including poor therapeutic response and lower survival. Anti-GSDMB antibody treatment can inhibit the growth, migration, and metastasis of breast cancer cells and enhance their sensitivity to trastuzumab [19]. GSDMB also promotes bladder carcinoma progression by activating the STAT3 pathway [29]. Rana et al. [30] found that in the CRC cell line HT29, full-length GSDMB could translocate to the membrane but did not induce cytotoxicity but instead promoted migration and proliferation. Conversely, our data showed that membranous GSDMB expression was somewhat correlated with lesser lymph node metastasis. Particularly, cytoplasmic GSDMB expression was related to a lower histological grade and reduced vessel infiltration, whereas nuclear GSDMB expression was associated with a lower histological grade. Unfortunately, IHC can only detect GSDMB expression at a certain time point but cannot reflect its dynamic expression. GSDMB in full-length or in cleavage may have different effects on cancer progression. Further investigations should be performed to reveal the mechanism behind these results on GSDMB expression.

Furthermore, we found that patients with cytoplasmic or nuclear GSDMB expression in cancer cells more often benefited from 5-Fu based chemotherapy. However, in cases that did not undergo chemotherapy, GSDMB expression did not affect prognosis, regardless of its location. In vitro assays in our study have indicated that GSDMB expression promoted 5-Fu–induced lytic cell death. A previous study also reported that 5-Fu–induced GSDME-mediated pyroptosis via caspase-3 activation in gastric cancer cells [31]. GSDMB has nuclear localization signals, and when it enters the nucleus, it can act either as a transcriptional coactivator or enhancer, but not as a transcription factor, to regulate the expression of many genes, such as TGF-β, ALOX5, and MMP9 [32]. Whether the relationship between nuclear GSDMB expression and chemotherapeutic efficacy is related to the downstream genes regulated by GSDMB remains unclear. The link between GSDMB and chemotherapeutic efficacy needs to be elucidated in further studies.

GSDM-mediated pore or pre-pore formation releases damage-related molecular patterns and cytokines, which directly modulate innate immune responses, increase antigen presentation and TLR activation, and lead to more extensive immune activation [33]. A low level of tumor cell pyroptosis caused by GSDMA3 induces effective antitumor immunity, including increased CD3^+^, CD4^+^, and CD8^+^ cell populations [24]. GSDME also mediates antitumor immunity depending on CD8^+^ lymphocytes and macrophages [34]. In patients with high GSDMB expression, the inhibitory effects on tumor are not only the direct killing of tumor by chemotherapy drugs, but also the further killing effect of immune cells on tumor. Through chemotherapy and various anti-tumor effects by immune cell, the prognosis of patients is ultimately improved. Our analyses of the correlations of membranous/cytoplasmic/nuclear GSDMB expression with CD3^+^/CD4^+^/CD8^+^ T lymphocytes, CD20^+^ B lymphocytes, CD68^+^ macrophages, and S100A8^+^ immune cells showed that membranous or nuclear GSDMB expression was positively correlated with S100A8^+^ immune cells in the TIF, whereas nuclear GSDMB was linked to a higher number of CD68^+^ macrophages in the entire tumor. S100A8 is expressed in myeloid lineage cells, including neutrophils and macrophages. Myeloid lineage cells are primary components of the innate immune system and play key roles in linking innate and adaptive immunity. Membranous GSDMB expression indicates pore formation and pyroptosis, inducing immune cell infiltration. The effects of nuclear GSDMB expression on the immune response may be related to its regulation of other genes, such as TGF-β, which regulates T cells, myeloid cells, and macrophages [32, 35]. Our previous studies have found that S100A8^+^ immune cells and CD68^+^ macrophages are favorable prognostic indicators in CRC [25, 36]. A limitation of our study is that we did not determine the different subtypes of macrophages.

In another study on GSDMB expression in CRC, Lu et al. [37] clustered TCGA-colon adenocarcinoma based on 12 pyroptosis-related regulators. The cluster with the highest GSDMB expression—which was linked to a higher number of activated CD8^+^ T cells, dendritic cells, macrophages, NK cells, and helper T cells—showed a better prognosis than the other cluster, which was related to advanced N stage. Of the 12 genes identified in that study, two were GSDMB and GSDMC, whereas the other genes were key regulators or markers of pyroptosis (such as GZMA, CASP3, IL1β). In our study, GSDMB expression was evaluated by IHC, which revealed a detailed staining pattern for identifying GSDMB expression in tumor and stromal cells, as well as to determine GSDMB expression in the tumor cell membrane, cytoplasm, and nucleus.

GSDMB has intricate relationships with many inflammatory diseases. GSDMB expression is increased in sepsis and Crohn’s disease [14], whereas GSDMB polymorphism is associated with asthma. A splice variant (rs11078928) in GSDMB skips an essential exon from the transcript, affects the induction of pyroptosis, and reduces the risk of asthma [13]. However, Das et al. [32] reported that GSDMB increased the risk of asthma in a murine model by regulating airway hyperresponsiveness and smooth muscle function without airway inflammation. In inflammatory bowel disease (IBD), GSDMB expression is upregulated to promote effective epithelial restitution and repair. However, the authors also pointed out that the GSDMB-dependent pyroptosis of intestinal epithelial cells could not be ruled out as a potential pathogenic mechanism during IBD [30]. In fact, the release of intracellular contents and pro-inflammatory mediators (e.g., IL-1β and IL-18) during pyroptosis can prompt systemic inflammation and a local immune reaction. Hence, we explored the association between GSDMB expression and systemic inflammatory parameters.

We found that membranous GSDMB expression was positively correlated with eosinophil count, whereas nuclear GSDMB expression was positively correlated with lymphocyte count and PNI. Conversely, cytoplasmic GSDMB expression was not correlated with any systemic inflammatory parameters in our study. The PNI, which was coined by Onodera et al. [38], is a predictor of the immunonutritional status of patients. Serum albumin is an important marker of nutritional status and non-specific inflammation, and peripheral lymphocyte count is a reflection of immune response. PNI is a prognostic factor in CRC [39]. More data are needed to establish the effect of GSDMB expression in cancer cells on systemic inflammation.

GSDM A to E are selectively expressed in different tissues, but all are expressed in the gastrointestinal tract. GSDMB is expressed in epithelial and immune cells. GSDMB in THP-1 cells could promote the cleavage of GSDMD and enhance non-canonical pyroptosis [14]. Notably, NK cells that eliminate cytosolic bacteria are regulated by GSDMB expression [11]. As far as we know, no reports have described the clinical significance of GSDMB expression in stromal immune cells in cancer. We found that GSDMB^+^ immune cell density was higher in younger and male patients but was not correlated with postoperative survival. Moreover, a high GSDMB^+^ immune cell density was linked to lower lymphocyte counts/percentage and monocyte counts/percentage, as well as to a higher neutrophil percentage in peripheral blood. Double immunofluorescence staining showed that GSDMB^+^ immune cells in cancer stroma were mostly CD68^+^ cells and S100A8^+^ cells.

## Conclusion

In conclusion, GSDMB is expressed in both cancer and immune cells, but its effects on cancer progression may be complex and possibly disparate. In cancer cells, GSDMB expression is linked to the systemic inflammatory response, TIM, and decreased cancer progression. Cytoplasmic GSDMB expression is an independent favorable prognostic indicator, whereas both cytoplasmic and nuclear GSDMB expression indicate improved chemotherapeutic efficacy in CRC. In immune cells, GSDMB expression is correlated with systemic inflammatory response. Our study provides evidence and insights for further research on GSDMB expression.

### Electronic supplementary material

Below is the link to the electronic supplementary material.

Supplementary Material 1

Supplementary Material 2

Supplementary Material 3

Supplementary Material 4

Supplementary Material 5

## Data Availability

The raw data are available on reasonable request from corresponding author.

## Abbreviations

GSDMB: Gasdermin B
CRC: Colorectal cancer
TIM: Tumor immune microenvironment
GSDM: Gasdermin
GSDMB-N: N-terminal domain of GSDMB
5-Fu: 5-Fluorouracil
TIF: Tumor invasive front
TC: Tumor center
LMR: Lymphocyte-to-monocyte ratio
NLR: Neutrophil-to-lymphocyte ratio
PLR: Platelet-to-lymphocyte ratio
PNI: Prognostic nutritional index
NOS: Not otherwise specified
IHC: Immunohistochemistry
DAB: 3, 3-Diaminobenzidine
PBS: Phosphate-buffered saline
HPF: High-power field
