# Supplementary figures and images for: Correlation of gasdermin B staining patterns with prognosis, progression, and immune response in colorectal cancer
Source: BMC Cancer. 2024 May 6;24:567. doi: 10.1186/s12885-024-12326-2 (PMC11075338; doi:10.1186/s12885-024-12326-2)

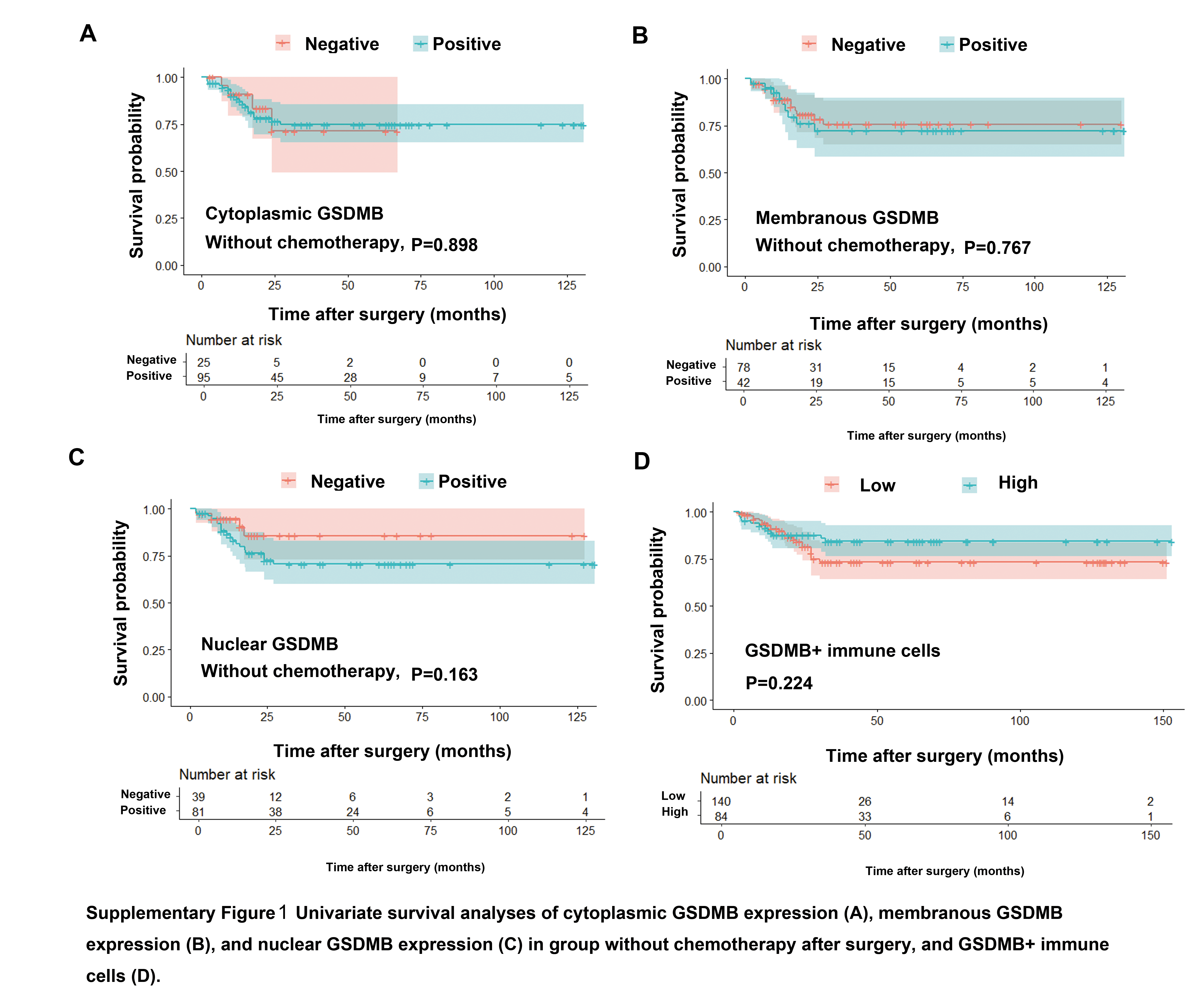

Supplement: Supplementary file 1 — Supplementary Material 1 [file 12885_2024_12326_MOESM1_ESM.tif]

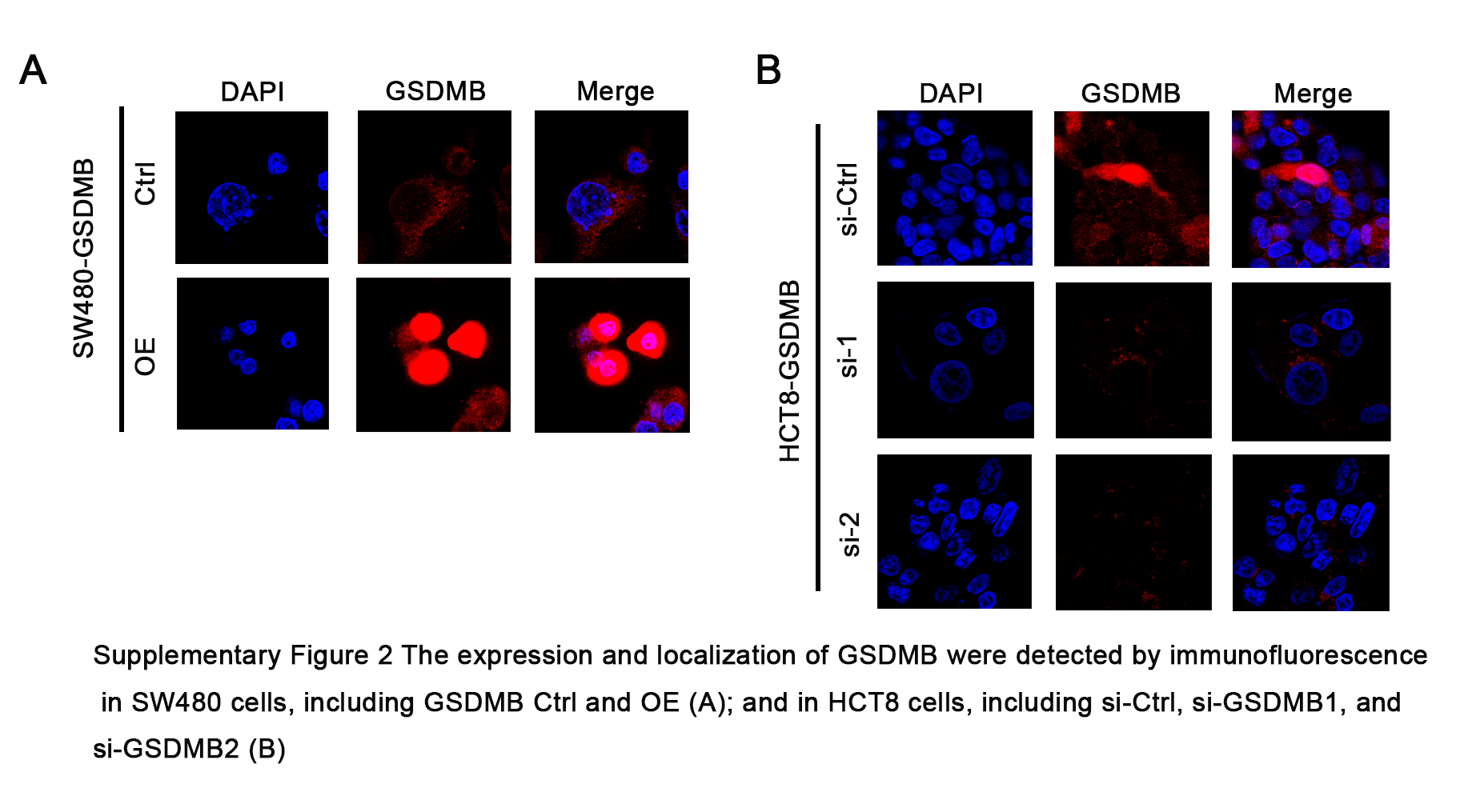

Supplement: Supplementary file 2 — Supplementary Material 2 [file 12885_2024_12326_MOESM2_ESM.tif]

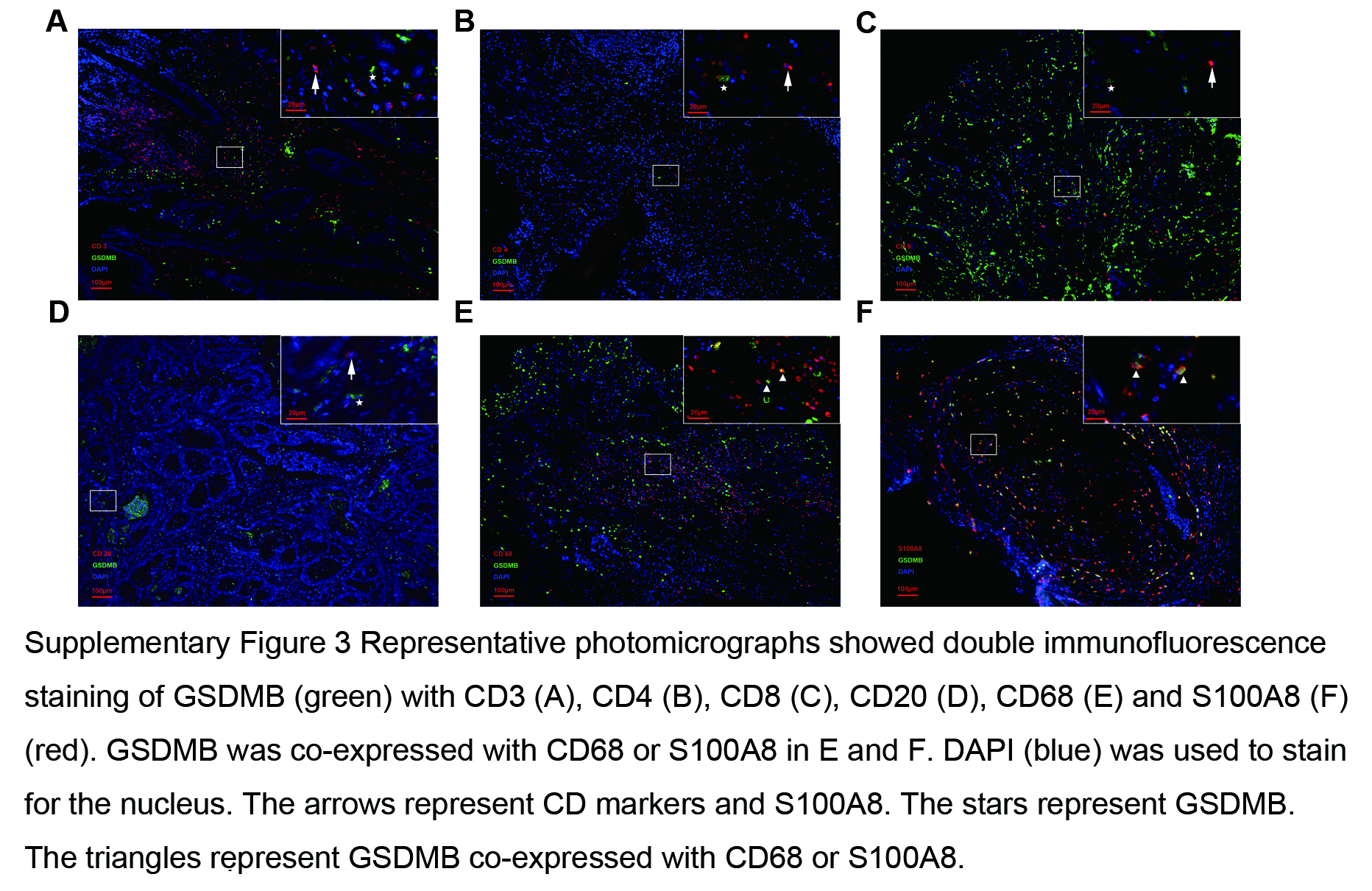

Supplement: Supplementary file 3 — Supplementary Material 3 [file 12885_2024_12326_MOESM3_ESM.tif]

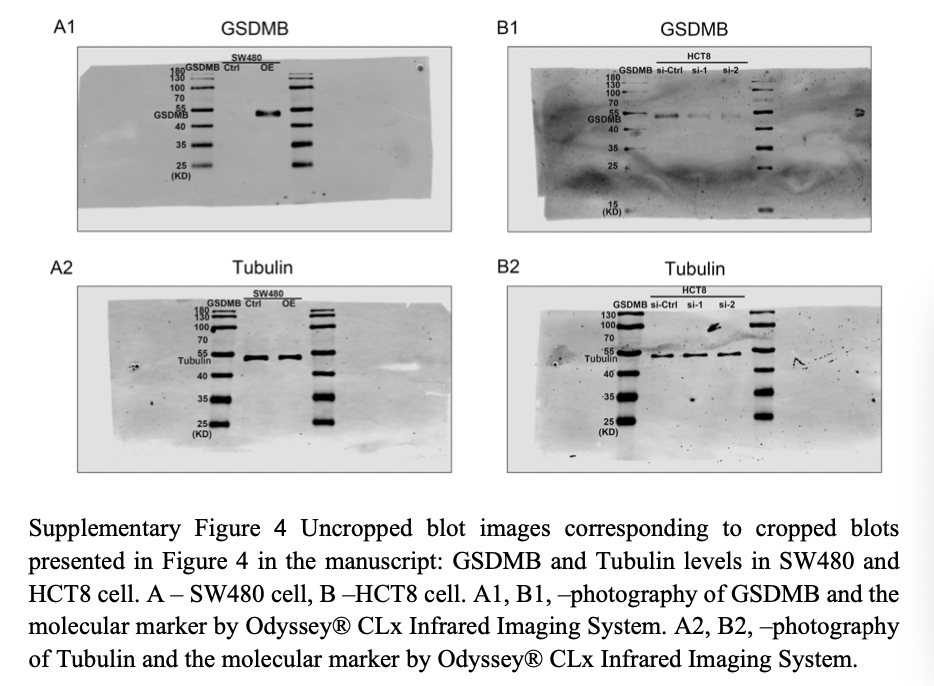

Supplement: Supplementary file 4 — Supplementary Material 4 [file 12885_2024_12326_MOESM4_ESM.tif]
