# Supplementary material for: Correlation of gasdermin B staining patterns with prognosis, progression, and immune response in colorectal cancer
Source: BMC Cancer. 2024 May 6;24:567. doi: 10.1186/s12885-024-12326-2 (PMC11075338; doi:10.1186/s12885-024-12326-2)
Supplement: Supplementary file 5 — Supplementary Material 5 [file 12885_2024_12326_MOESM5_ESM.docx]

Supplementary Table 1 Information of immunohistochemical staining

| Antibody | Source | Dilution | Staining pattern |
| --- | --- | --- | --- |
| GSDMB | Feng Shao’s Lab (Beijing, China) | 1:250 | Membrane/Cytoplasmic/ Nucleus |
| CD3 | Thermo Fisher, Waltham, MA, USA | 1:300 | Cytoplasmic |
| CD4 | ZSGB-BIO, Beijing, China | 1:100 | Cytoplasmic |
| CD8 | Thermo Fisher, Waltham, MA, USA | 1:50 | Cytoplasmic |
| CD20 | Thermo Fisher, Waltham, MA, USA | 1:500 | Cytoplasmic |
| CD68 | DAKO, Copenhagen, Denmark | 1:200 | Cytoplasmic |
| S100A8 | Santa Cruz, Dallas, TX, USA | 1:100 | Cytoplasmic |

Supplementary Table 2 The consistency between GSDMB expression in tumor cells and in normal epithelial cells

| GSDMB | | | In tumor cells | | | | | | | | | | | |
| --- | --- | --- | --- | --- | --- | --- | --- | --- | --- | --- | --- | --- | --- | --- |
|  |  |  | Total | | *P* value | Membrane | | *P* value | Cytoplasm | | *P* value | Nucleus | | *P* value |
|  |  |  | - | + |  | - | + |  | - | + |  | - | + |  |
| In normal epithelial cells | Total | - | 11(37.9) | 5(301) | **<0.011*** |  |  |  |  |  |  |  |  |  |
|  |  | + | 18(62.1) | 154(96.9) |  |  |  |  |  |  |  |  |  |  |
|  | Membrane | - |  |  |  | 108(78.8) | 30(58.8) | 1.000 |  |  |  |  |  |  |
|  |  | + |  |  |  | 29(21.2) | 21(41.2) |  |  |  |  |  |  |  |
|  | Cytoplasm | - |  |  |  |  |  |  | 17(41.5) | 19(12.9) | 0.542 |  |  |  |
|  |  | + |  |  |  |  |  |  | 24(58.5) | 128(87.1) |  |  |  |  |
|  | Nucleus | - |  |  |  |  |  |  |  |  |  | 15(31.3) | 10(7.1) | **0.001*** |
|  |  | + |  |  |  |  |  |  |  |  |  | 33(68.8) | 130(92.9) |  |

Data presented as the number (percentage) of cases with positive GSDMB staining

Significant *P* values are indicated with asterisks

Supplementary Table 3 Association between total GSDMB expression in cancer cells and clinicopathological indicators

| Clinicopathological  indicators | numbers | GSDMB expression (%) | | *P* value |
| --- | --- | --- | --- | --- |
|  |  | Negative | Positive |  |
| Age |  |  |  | 0.597 |
| ≤60 years old | 94 | 16(39.0) | 78(34.5) |  |
| >60 years old | 173 | 25(61.0) | 148(65.5) |  |
| Sex |  |  |  | 0.499 |
| Male | 144 | 20(48.8) | 124(54.9) |  |
| Female | 123 | 21(51.2) | 102(45.1) |  |
| Location |  |  |  | 0.612 |
| Colon | 135 | 19(46.3) | 116(51.3) |  |
| Rectum | 132 | 22(53.7) | 110(48.7) |  |
| Histologic type |  |  |  | 0.171 |
| adenocarcinoma NOS others | 200  67 | 27（65.9）  14（34.1） | 173（76.5）  53（23.5） |  |
| Histological grade |  |  |  | **0.040*** |
| Low | 188 | 23(56.1) | 165(73.0) |  |
| High | 79 | 18(43.9) | 61(27.0) |  |
| Perineural infiltration |  |  |  | 0.714 |
| Absent | 184 | 27(65.9) | 157(69.5) |  |
| Present | 83 | 14(34.1) | 69(30.5) |  |
| Vessel infiltration |  |  |  | 0.053 |
| Absent | 229 | 31（75.6） | 198（87.6） |  |
| Present | 38 | 10（24.4） | 28（12.4） |  |
| Infiltration depth |  |  |  | 0.858 |
| Within serosa | 90 | 13(31.7) | 77(34.1) |  |
| Outside serosa or muscle | 177 | 28(68.3) | 149(65.9) |  |
| Lymph node metastasis |  |  |  | 0.399 |
| Absent | 141 | 19(46.3) | 122(54.0) |  |
| Present | 126 | 22(53.7) | 104(46.0) |  |
| Distant metastasis |  |  |  | 0.568 |
| Absent | 241 | 36(87.8) | 205(90.7) |  |
| Present | 26 | 5(12.2) | 21(9.3) |  |
| TNM stage |  |  |  | 0.926 |
| I | 57 | 8(19.5) | 49(21.7) |  |
| II | 78 | 11(26.8) | 67(29.6) |  |
| III | 106 | 17(41.5) | 89(39.4) |  |
| IV | 26 | 5(12.2) | 21(9.3) |  |

Significant *P* values are indicated with asterisks

Supplementary Table 4 Association between the GSDMB^+^ immune cell density in TIF and clinicopathological indicators

| clinicopathological indicators | number | GSDMB^+^ immune cells (%) | | *P* value |
| --- | --- | --- | --- | --- |
|  |  | Low | High |  |
| Age |  |  |  | **0.014*** |
| ≤60 years old | 78 | 40(28.6) | 38 (44.7) |  |
| >60 years old | 147 | 100(71.4) | 47(55.3) |  |
| Sex |  |  |  | **0.006*** |
| Male | 122 | 66(47.1) | 56(65.9) |  |
| Female | 103 | 74(52.9) | 29(34.1) |  |
| Location |  |  |  | 0.250 |
| Colon | 116 | 68(48.6) | 48(56.5) |  |
| Rectum | 109 | 72(51.4) | 37(43.5) |  |
| Histologic type |  |  |  | 0.374 |
| Adenocarcinoma NOS | 170 | 103（73.6） | 67（78.8） |  |
| Others | 55 | 37（26.4） | 18（21.2） |  |
| Histological grade |  |  |  | 0.951 |
| Low | 162 | 101(72.1) | 61(71.8) |  |
| High | 63 | 39(27.9) | 24(28.2) |  |
| Perineural infiltration |  |  |  | 0.984 |
| Absent | 156 | 97 (69.3) | 59(69.4) |  |
| Present | 69 | 43(30.7) | 26(30.6) |  |
| [Vessel](javascript:;) infiltration |  |  |  | 0.720 |
| Absent | 193 | 121（86.4） | 72（84.7） |  |
| Present | 32 | 19（13.6） | 13（15.3） |  |
| Infiltration depth |  |  |  | 0.596 |
| Within serosa | 72 | 43 (30.7) | 29(34.1) |  |
| Outside serosa or muscle | 153 | 97(69.3) | 56(65.9) |  |
| Lymph node metastasis |  |  |  | 0.815 |
| Absent | 124 | 78(55.7) | 46(54.1) |  |
| Present | 101 | 62(44.3) | 39(45.9) |  |
| Distant metastasis |  |  |  | 0.543 |
| Absent | 207 | 130(92.9) | 77(90.6) |  |
| Present | 18 | 10(7.1) | 8(9.4) |  |
| TNM stage |  |  |  | 0.823 |
| I | 48 | 29(20.7) | 19(22.4) |  |
| II | 71 | 47(33.6) | 24(28.2) |  |
| III | 88 | 54(38.6) | 34(40.0) |  |
| IV | 18 | 10(7.1) | 8(9.4) |  |

Significant *P* values are indicated with asterisks
